# Influence of SiC and ZnO Doping on the Electrical Performance of Polylactic Acid-Based Triboelectric Nanogenerators

**DOI:** 10.3390/s24082497

**Published:** 2024-04-13

**Authors:** Stefania Skorda, Achilleas Bardakas, George Vekinis, Christos Tsamis

**Affiliations:** Institute of Nanoscience and Nanotechnology (INN), National Centre for Scientific Research “Demokritos”, Patr. Gregoriou E & 27 Neapoleos Str., Aghia Paraskevi, 15310 Athens, Greece; stef.skorda@gmail.com (S.S.); a.bardakas@inn.demokritos.gr (A.B.); g.vekinis@inn.demokritos.gr (G.V.)

**Keywords:** tribogenerators, energy harvesting, polylactic acid, zinc oxide, silicon carbide, 3D printing

## Abstract

Polylactic acid (PLA) is one of the most widely used materials for fused deposition modeling (FDM) 3D printing. It is a biodegradable thermoplastic polyester, derived from natural resources such as corn starch or sugarcane, with low environmental impact and good mechanical properties. One important feature of PLA is that its properties can be modulated by the inclusion of nanofillers. In this work, we investigate the influence of SiC and ZnO doping of PLA on the triboelectric performance of PLA-based tribogenerators. Our results show that the triboelectric signal in ZnO-doped PLA composites increases as the concentration of ZnO in PLA increases, with an enhancement in the output power of 741% when the ZnO concentration in PLA is 3 wt%. SiC-doped PLA behaves in a different manner. Initially the triboelectric signal increases, reaching a peak value with enhanced output power by 284% compared to undoped PLA, when the concentration of SiC in PLA is 1.5 wt%. As the concentration increases to 3 wt%, the triboelectric signal reduces significantly and is comparable to or less than that of the undoped PLA. Our results are consistent with recent data for PVDF doped with silicon carbide nanoparticles and are attributed to the reduction in the contact area between the triboelectric surfaces.

## 1. Introduction

Triboelectric energy harvesting has emerged as a very important alternative for the conversion of the abundant mechanical energy that surrounds us to electrical energy for powering electronic devices and systems. Following the introduction of the triboelectric nanogenerator (TENG) by the group led by Zhong Lin Wang [1], there has been a rapid increase at the number of published works on triboelectric harvesting using a variety of materials and technologies [2,3,4,5,6]. This is because triboelectrification and electrostatic induction, which underlie the operation of TENGs, can occur for almost all material combinations, the challenge being to identify the material pairs that will maximize the triboelectric signal. With the establishment of 3D printing technologies [7,8] as low-cost manufacturing processes able to produce a variety of structures with minimal material waste and reduced manufacturing time, it is not surprising that 3D printing-based triboelectric devices have been developed for self-powered electronics [9].

Polylactic acid (PLA) is one of the natural thermoplastic materials that has been utilized for the fabrication of triboelectric devices. PLA is a recyclable, biocompatible and biodegradable polyester that is produced from renewable resources and has been widely used in the food industry as well as for medical and electronic applications.

Qiao et al. [10] investigated fused deposition modeling (FDM) for the fabrication of triboelectric energy harvesters. Several positive and negative polymers were evaluated as friction layers, including polylactic acid (PLA), nylon (PA), etc., to achieve a satisfactory output power and a high energy conversion efficiency. A fully biodegradable triboelectric nanogenerator based on a gelatin film and an electrospun PLA nanofiber membrane has been presented by Pan et al. [11]. By optimizing the material properties, they achieved an output voltage up to 500 V, a short circuit current density of 10.6 mA/m^2^, and a maximum power density greater than 5 W/m^2^.

One of the techniques used to improve the performance of the triboelectric nanogenerators is dielectric layer doping [12]. This can be achieved by the introduction of dopants with a high dielectric constant in the dielectric layer to increase the capacitance/dielectric constant of the composite material without altering its physical properties. Zhang et al. [13] demonstrated an endogenous TENG based on a PLA composite fiber film using MXene as the matrix and filler, respectively. They showed that the open-circuit voltage, short-circuit current, and charge are increased when PLA–MXene contacts were introduced between the fibers, reaching the highest values of 16 V, 1.2 mA, and 7 nC, respectively, in the case of 1 wt% MXene/PLA (1 wt% PMX). Shi et al. [14] doped PLA with poly(ethylene glycol) monomethyl ether (mPEG) for fiber fabrication using the electrospinning technique. They showed that the incorporation of mPEG enhanced the electron-donating ability and charge transfer efficiency in PLA while the PLA/mPEG-based TENGs achieved an open-circuit voltage of 342.8 V, a short-circuit current of 38.5 μA, and a maximum power density of 116.21 W m^−2^. Li et al. [15] utilized a rotating, freestanding mode TENG, where the rotor was made of polylactide (PLA) material to evaluate a power regulation circuit (PRC) suitable for TENGs, which is composed of a valley-filling circuit and a switching step-down circuit.

In this work, we exploit dielectric layer doping to enhance the triboelectric output of 3D-printed tribogenerators. Various concentrations of SiC and ZnO inclusions are incorporated in PLA-based tribogenerators and their influence on the performance of TENGs is evaluated.

## 2. Materials and Methods

### 2.1. Materials

PLA pellets (Luminy LX175), used as the matrix for the composite ma-terial, were obtained from Total Corbion (Gorinchem, The Netherlands). SiC powder (#1200 mesh, about 8.3 μm grain size) was supplied by Struers (Copenhagen, Denmark). ZnO powder (≤100 nm in particle size) was provided by Sigma-Aldrich (Merck KGaA, Darmstadt, Germany).

### 2.2. Preparation of Filament and Samples

The preparation of the SiC-doped PLA composites has been reported in [16]. In brief, after drying at 100 °C for 24 h, the PLA pellets were mixed with SiC powder at 75 °C with the addition of acetone. The final mixture was dried again at 100 °C for 24 h and stored in a desiccator until extrusion. Five composites’ concentrations were prepared with concentrations ranging from 1 wt% to 3 wt%. Using a single-screw extruder (Felfil Evo, Felfil, Turin, Italy) a continuous composite filament, 1.75 mm in diameter, was produced for each concentration. Pure PLA filament was also produced with identical conditions. Finally, three ZnO-doped PLA composites were prepared with a similar method, with concentrations ranging from 1 wt% to 3 wt%.

### 2.3. Tribogenerators

The triboelectric nanogenerators used in this study operate according to the contact-separation mode. Circular samples with a diameter of 32 mm were 3D printed using a CREALITY CR20 Pro 3D printer (Creality 3D Technology Co., Ltd., Shenzhen, China) and the aforementioned doped PLA filaments. The specimens that were used in this study were fabricated with the produced filaments. A grinder/polisher (STRUERS DAP-7, Struers, Copenhagen, Denmark) was used to polish the 3D printed samples using a SiC polishing paper with mesh size #1000 until they reached a thickness of about 390 μm. A constant water supply was used to eliminate any remaining particles. Following that, the samples were cleaned with absolute alcohol and dried.

**Figure 1:**
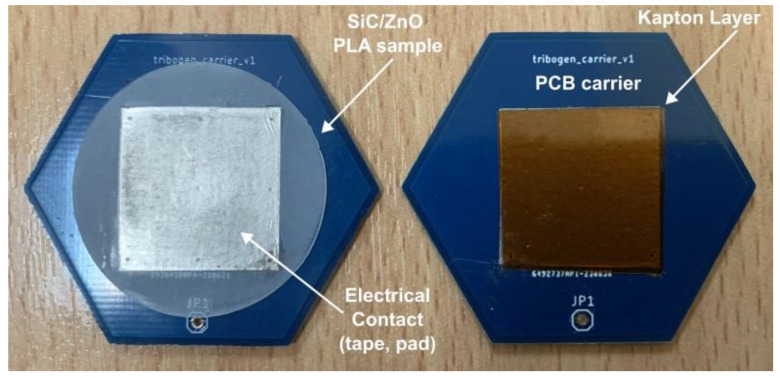
PCB carrier for mounting the fabricated materials, and the Kapton films used as the reference surface, for the triboelectric characterization.

As a reference triboelectric surface, a 75 μm-thick Kapton^®^ layer (DuPont™, Kingston, ON, Canada) was cut from a large sheet to a dimension of 2 × 2 cm^2^. All triboelectric surfaces were mounted on hexagonal PCB sample carriers using double-sided aluminum conductive tape, completing a triboelectric pair, shown in Figure 1. The PCB carrier boards contain a contact pad on the backside providing the electrical contact for the triboelectric surfaces.

In addition, circular capacitors were fabricated in order to estimate the change in the dielectric constant as a function of ZnO doping concentration. Circular samples of 32 mm were 3D printed using the abovementioned prepared filaments. The measured thickness for all samples is given in Table 1. Following printing, the samples were polished and 200 nm Al was deposited on both sides using a shadow mask to form a circular plate capacitor of 25 mm in diameter.

### 2.4. Characterization of PLA and Doped PLA

Morphological and elemental characterization of the fabricated composites was performed using scanning electron microscopy (SEM, FEI Quanta Inspect, Thermo Fisher Scientific Inc, Waltham, MA, USA) and energy dispersive X-ray spectroscopy (EDX, 10 keV). The topography and surface roughness of the fabricated samples doped with SiC and ZnO after polishing were analyzed via white light interferometry (WLI) using a 3D optical profilometer (Profil3D^®^, Filmetrics, San Diego, CA, USA).

Capacitance measurements were performed using an HP 4284A LCR meter (Hewlett-Packard, Palo Alto, CA, USA). Measurements were performed for frequencies ranging from 1 kHz to 1 MHz and for biases ranging from 0 V to ±2 V. From the capacitance measurements, the dielectric constant was extracted using the following equation:

(1) $$C_{x}=\frac{\varepsilon_{ο}\cdot \varepsilon_{x}\cdot A}{d}$$

where C_x_ is the measured capacitance, ε_o_ = 8.854 × 10^−12^ CV^−1^ m^−1^ is the dielectric permittivity of vacuum, ε_x_ is the composite material dielectric constant, A is the capacitor area, and d is the material thickness.

### 2.5. Characterization of Tribogenerators

**Figure 2:**
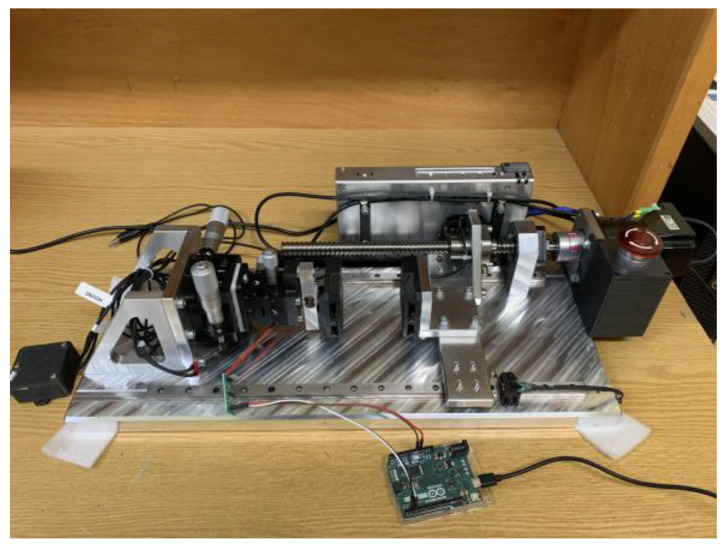
The in-house experimental setup used for the triboelectric characterization of the produced pure PLA, SiC-doped PLA, and ZnO-doped PLA samples at contact-separation mode.

To investigate the output performance of PLA-based TENGs, three types of measurements were performed: (a) time-dependent output voltage (transient), (b) open circuit voltage (V_oc_), and (c) short circuit current (I_sc_). For the transient measurements, an InfiniiVision DSO7104A oscilloscope (Agilent Technologies, Santa Clara, CA, USA) was used. Current measurements were performed with a Stanford Research Systems SR570 (Stanford Research Systems, Sunnyvale, CA, USA) low-noise current preamplifier. For the estimation of the power as a function of an external load, as well as the open circuit voltage, the methodology developed by Jayasvasti et al. [17] was applied. Those researchers have demonstrated that by adding a 1 MΩ resistance in parallel with the oscilloscope probe (input resistance 10 MΩ) during the measurements of a TENG, more precise results can be obtained for the extraction of the power output compared to the typical approach where the TENG voltage is measured directly across the load. For the periodic motion of the triboelectric surfaces, the in-house system shown in Figure 2 was used. A servo motor (PD4-CB59M024035-E-01 DC, Nanotec Electronic GmbH & Co. KG, Feldkirchen, Germany) was used, in conjunction with a ballscrew, to establish a linear motion between the two triboelectric surfaces, with one of them being stationary and the other moving. The velocity and acceleration of the motion were controlled by a computer and were maintained constant for all experiments.

## 3. Results and Discussion

### 3.1. Structural Characterization

**Figure 3:**
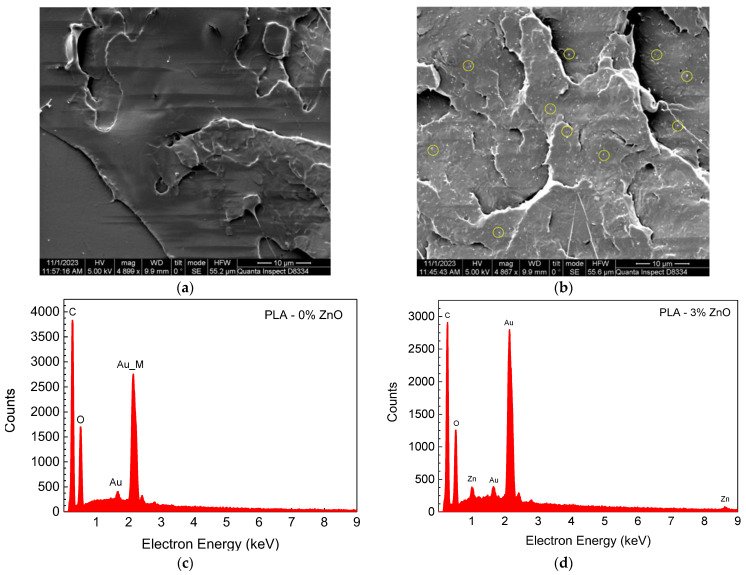
SEM micrograph of (**a**) undoped PLA and (**b**) 3 wt% ZnO-doped PLA (the yellow circles indicate the presence of the ZnO nanoparticles) and EDX spectrum for (**c**) undoped PLA and (**d**) 3 wt% ZnO-doped PLA.

Figure 3a is an SEM micrograph of the undoped PLA samples along a fractured surface, while Figure 3b is an SEM micrograph for the 3% ZnO-doped PLA samples. By comparing the two photographs, we can identify the ZnO nanoparticles as white dots in Figure 3b, while no such distribution is seen in Figure 3a. The distribution of the ZnO nanoparticles appears to be uniform. The presence of ZnO nanoparticles was also confirmed by EDX analysis of the samples. Figure 3c is an EDX spectrum for the undoped PLA, while Figure 3d shows the spectrum for 3 wt% ZnO-doped PLA. In Figure 3d, the L and K emission peaks of Zn at 1.011 keV and 8.61 keV are clearly identified, which are not observed in Figure 3c, verifying the existence of ZnO nanoparticles in the doped samples. The emission peaks at 1.66 eV and 2.14 eV correspond to the thin gold layer that was deposited on the PLA sample prior to SEM imaging, in order to avoid surface charging phenomena. The analysis (SEM, EDX) of the SiC-doped PLA is presented in detail in reference [16].

### 3.2. Electrical Characterization—Estimation of Dielectric Constant

**Figure 4:**
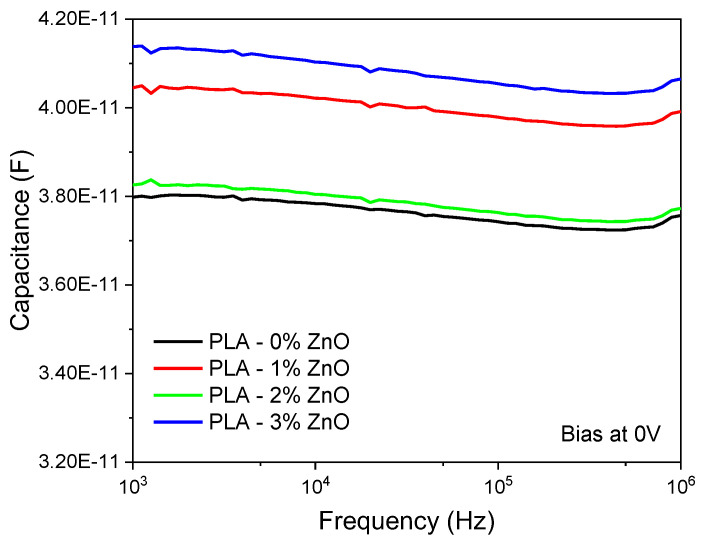
Capacitance vs. frequency at zero bias voltage for all samples.

Figure 4 shows the capacitance as a function of the frequency at zero bias for the ZnO-doped samples. We notice that the capacitance is almost constant for all frequencies, with small variations that can be attributed to Al/PLA interface charges. The variation in the capacitance values can be attributed to variations in capacitor thickness (Table 1) as well as the dielectric constant.

**Figure 5:**
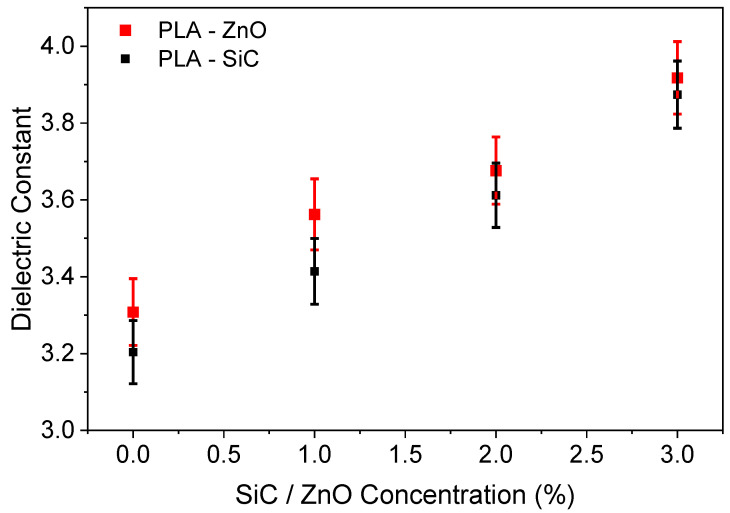
Dielectric constant of the PLA–ZnO composite material as a function of ZnO concentration.

Taking the measured thickness values (Table 1) and the capacitance values at 10 KHz into account, we can calculate, by using Equation (1), the dielectric constant as a function of ZnO doping. The results are shown in Figure 5. We notice that the dielectric constant of the ZnO-doped PLA increases monotonically from 3.3 for undoped PLA to 3.9 for 3%ZnO-doped PLA. Such behavior has been reported in the literature when materials of various shapes are immersed in a matrix [18] if the dielectric constant of the inclusions is higher compared to the host matrix. This applies to our case, since the dielectric constant of ZnO nanoparticles is about 10 [19], much higher compared to the dielectric constant of PLA, which ranges from 2.5 to 3.11 [20,21]. In the same figure, we have also included measurements for the SiC-doped samples from our previous work [16], where a similar increase in the dielectric constant of SiC-doped PLA can be observed.

### 3.3. SiC-Based Tribogenerators

**Figure 6:**
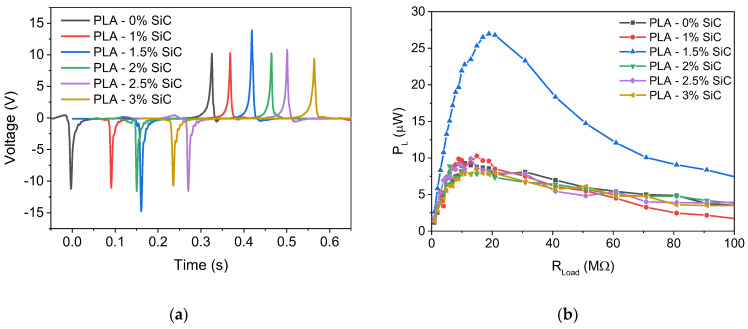
(**a**) Time-dependent triboelectric voltage signal and (**b**) power as a function of the external load for tribogenerators with different SiC concentrations.

Figure 6a shows the triboelectric signal as a function of time for the various TENGs doped with SiC. We notice that initially the triboelectric signal increases from 11.3 V for undoped PLA to 14.4 V for PLA doped with 1.5 wt% SiC. However, as the SiC concentration increases to 3 wt%, the triboelectric signal is reduced to 10.5 V, which is comparable to the value obtained for the undoped PLA. Similar results can be extracted from Figure 6b where the dependence of the generated power is shown as a function of the external load. We notice that the maximum power obtained for the 1.5 wt% SiC-doped PLA is 26 μW (6.5 μW/cm^2^, considering that the surface area of the samples is 4 cm^2^), which presents a 2.5 times increase compared to all other samples.

**Figure 7:**
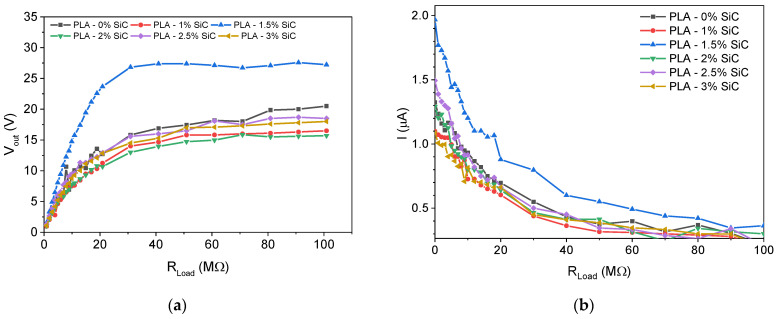
(**a**) Open circuit voltage (V_oc_) and (**b**) short circuit current (I_sc_) for tribogenerators with different SiC concentration.

Figure 7a shows the voltage output as a function of the external load. We notice that the 1.5 wt% SiC-doped PLA exhibits the highest open circuit voltage reaching a value of 26 V. A similar result is obtained from Figure 7b, where the tribogenerator current is shown as a function of the external load. We observe that the short circuit current (at zero external load) for the 1.5 wt% SiC-doped PLA reaches 2 μA (500 nA/cm^2^), which is substantially higher compared to all other SiC-doped samples.

### 3.4. ZnO-Based Tribogenerators

**Figure 8:**
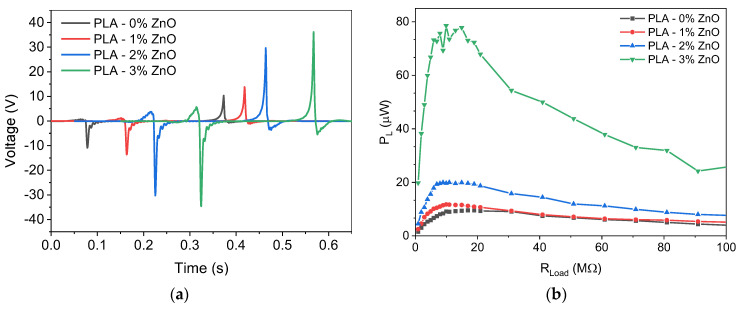
(**a**) Transient signal as a function of time and (**b**) power as a function of the external load for tribogenerators with different ZnO concentrations.

Figure 8a shows the triboelectric signal as a function of time for the various ZnO-doped PLA TENGs. We notice that the triboelectric signal increases as the concentration of ZnO increases, reaching a value of 40 V when the concentration of ZnO is 3 wt%. Similar results can be extracted from Figure 8b where the dependence of tribogenerator power is shown as a function of the external load. We notice that the maximum power obtained for the 3 wt% ZnO-doped PLA is 80 μW (20 μW/cm^2^), which is about eight times higher compared to the undoped PLA.

**Figure 9:**
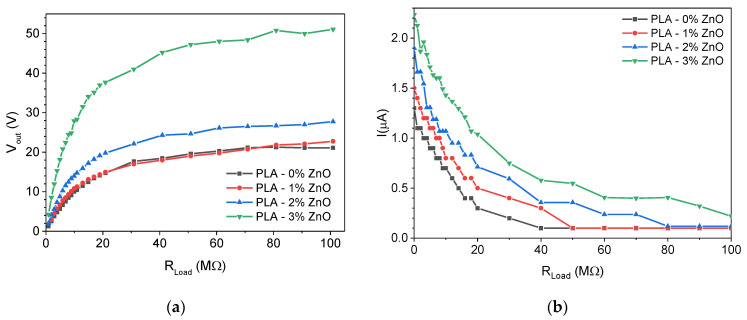
(**a**) Open circuit voltage (V_oc_) and (**b**) short circuit current (I_sc_) for tribogenerators with different ZnO concentrations.

Figure 9a shows the voltage output as a function of the external load. We notice that the 3 wt% doped ZnO exhibits the highest open circuit voltage reaching a value of 50 V. Similar results can be obtained from Figure 9b, where the tribogenerator current is shown as a function of the external load. We notice that the short circuit current for the 3 wt% ZnO-doped PLA reaches 2.2 μA (550 nA/cm^2^), which is higher compared to all other samples.

### 3.5. Analysis of the Results

**Figure 10:**
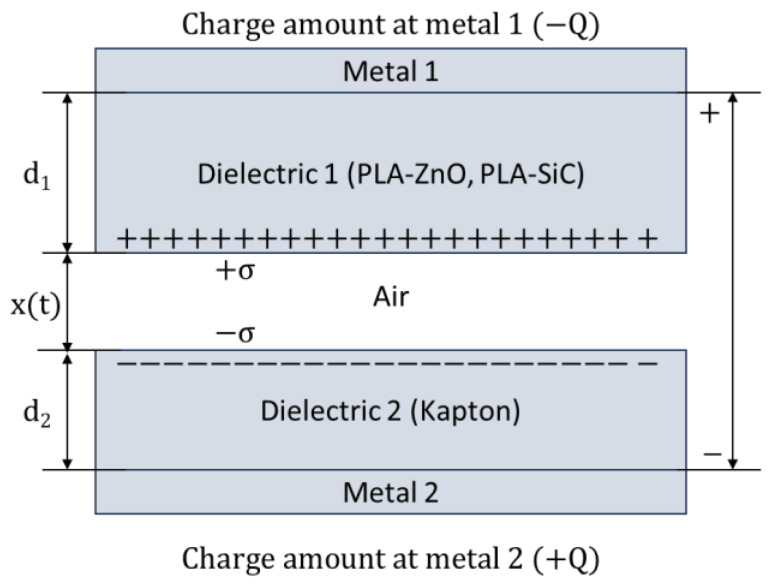
Schematic of the triboelectric generator in contact-separation mode.

To better understand the results, we refer to the theoretical analysis of Niu et al. [22]. Figure 10 shows a schematic of a tribogenerator composed of two dielectric surfaces and the electrodes attached at the back side of the dielectrics. The TENG operates in the contact-separation mode. When the two triboelectric surfaces are in contact, electric charges of opposite polarities appear on the surfaces of the dielectrics, due to charge transfer between the two materials. In our case, the surface of PLA is charged positively whereas the surface of Kapton is charged negatively, due to the differences in the electronegativities of the two materials.

The voltage that develops across the device is given by the V–Q–x relationship

(2) $$V(t)=-\frac{Q}{{S\varepsilon }_{0}}\left[d_{0}+x\left(t\right)\right]+\frac{\sigma x\left(t\right)}{\varepsilon_{0}}$$

where Q is the amount of charge at the metal contact, σ is the surface charge density on the surface of the dielectrics, ε_0_ is the permittivity of vacuum, S is the contact area between the triboelectric pair, and d_0_ is given by the equation

(3) $$d_{0}=\frac{d_{1}}{\varepsilon_{r1}}+\frac{d_{2}}{\varepsilon_{r2}}$$

where d_1_, d_2_ and ε_r1_, ε_r2_ are the thicknesses and relative dielectric constants of the dielectric layers 1 and 2, respectively. In our case, where Kapton^®^ is used as the reference electrode and composite PLA is used as the active electrode the values are d_1_ = 75 μm, ε_r1_ = 3, and d_2_ = 390 μm. The value for ε_r2_ depends on the composite doping.

Alternatively, Equation (1) can be written in the form

(4) $$V(t)=-\frac{Q}{C_{TENG}}+V_{oc}$$

where C_TENG_ is the total capacitance of the TENG given by the Equation (4)

(5) $$C_{TENG}=\frac{{S\varepsilon }_{0}}{\left[d_{0}+x\left(t\right)\right]}$$

and V_oc_ is the open circuit voltage as defined in Equation (5)

(6) $$V_{oc}=\frac{\sigma \cdot x(t)}{\varepsilon_{o}}$$

Figure 9 shows a comparison of the open circuit voltage V_oc_ and the short circuit current I_sc_ of the TENGs with the various concentrations of SiC and ZnO. We see that as the concentration of SiC and ZnO increases, V_oc_ and I_sc_ are enhanced in both cases. In fact, for the SiC-doped PLA, V_oc_ and I_sc_ are increased by 42% and 48%, respectively, compared to undoped PLA as the concentration increases to 1.5 wt%, while for ZnO-doped PLA the increase is 44% and 69%, respectively, as the concentration increases to 2%. A similar behavior is observed for the maximum power with the increase being 280% and 188%, respectively.

Such a behavior can be attributed to the improvement of the capacitance/dielectric constant of doped PLA due to the introduction of fillers (micro/nanoparticles) with higher dielectric constants compared to the PLA [12,23]. It has been demonstrated that the inclusion of fillers within a dielectric can enhance the triboelectric signal. This is because nanoparticles with higher dielectric constants compared to the host, when dispersed in the matrix, will act as microcapacitors. Upon the presence of an electric field, charge will accumulate at the interface of the particles, leading to interfacial polarization and an enhancement of the surface charge density of the dielectric layer [12].

**Figure 11:**
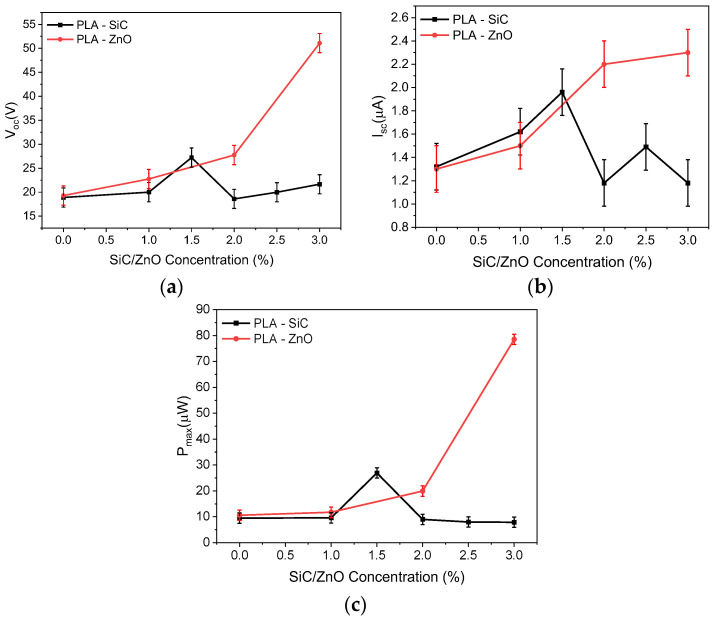
(**a**) Open circuit voltage, (**b**) short circuit current, and (**c**) power of the TENGs with the various concentrations of SiC and ZnO.

Moreover, the presence of these particles will increase the dielectric properties of the composite material, as we have shown in Section 3.2. This increase in the dielectric constant significantly increased the density of the charges that can be accumulated on the copolymer during physical contact [24]. From Figure 11a, and considering Equation (5), we can conclude that the surface charge density for the 1.5 wt% SiC-doped samples and the 3 wt% ZnO-doped samples is increased compared to the undoped PLA, reaching a value of 2.65 μC/m^2^ and 4.86 μC/m^2^, respectively.

Another point that we notice from Figure 11 is the difference in behavior between the PLA-doped materials. We observe that for the ZnO-doped samples the triboelectric signal continues to increase as the concentration increases to 3 wt%. In fact, the open circuit voltage, short circuit current, and power output are increased by 265%, 177%, and 741%, respectively, compared to the undoped PLA.

However, the SiC-doped PLA behaves in a different way. The triboelectric signal reaches a maximum (at 1.5 wt% in our case) and then it is reduced as the concentration is increased to 3 wt%. To better comprehend this behavior, we take the mechanisms that influence the triboelectric signal into consideration. For the doped PLA samples, there are two competing mechanisms that determine the triboelectric signal: (a) the doping with the SiC ot ZnO particles, which lead to an increase in the triboelectric signal due to increased dielectric constant/capacitance of the doped PLA samples, as we have analysed above; and (b) an increase in surface roughness, which leads to a decrease in the triboelectric signal due to the reduction in the active contact area between the two triboelectric surfaces. As we have mentioned, during preparation the samples were polished with a SiC polishing paper to reduce the surface roughness of the 3D printed surface. This results in a relatively low value of surface roughness. From optical profiler measurements, we estimate the root mean square height is less than 0.3 μm. However, as the concentration of SiC in the PLA matrix to increases 3 wt%, the surface roughness increases by 33%, reaching a value of 0.41 μm. This results in a reduction in the surface contact area leading to a decrease in the triboelectric signal. In contrast, for the ZnO-doped PLA, optical profiler measurements indicate that the incorporation of ZnO particles in the PLA does not influence the surface roughness. This can be attributed to the smaller size of ZnO nanoparticles as well as the fact that ZnO is much softer compared to the SiC grinding paper.

The reduction in the triboelectric signal due to the increased surface roughness is in agreement with literature results. Kumar et al. [25] investigated the impact of random multiscale surface roughness on the triboelectric signal of TENGs. These researchers developed TENGs based on transparent mica in contact with polyvinyl siloxane (PVS) with modulated surface roughness. With the aid of a novel in situ optical technique, they were able to directly estimate the contact surface area as a function of the roughness. They observed that, as the roughness increased, the triboelectric signal as well as the output performance of the TENG did as well, due to the decrease in the real contact area. Similar results were obtained from Wen et al. [26] who investigated, both theoretically and experimentally, a conductor–dielectric contact separation TENG for sensing the roughness of material surfaces. The reduction in the triboelectric signal for PVDF-doped SiC has also been reported in the literature by Shafeek et al. [27]. Those researchers investigated the influence of SiC nanoparticles on the triboelectric properties of a polyvinylidene fluoride (PVDF)/silicon carbide (SiC) nanocomposite. They showed that the triboelectric signal can be greatly enhanced compared to pristine PVDF as the concentration of SiC increased up to 6%. However, for higher concentrations (9%) the device output tends to decrease. They attributed this decrease to the agglomeration of SiC nanoparticles on the surface of the PVDF films resulting in reduced contact area between the PVDF and the polyamide that was used as a reference material.

## 4. Conclusions

In this work, we investigated the influence of SiC microparticles and ZnO nanoparticles on the triboelectric performance of PLA-doped tribogenerators. In both cases, an improved performance was observed due to the higher dielectric constant of the dopants compared to the undoped PLA matrix. For the SiC microparticles, a maximum value for the open circuit voltage, short circuit current, and maximum power output was observed for a doping concentration of 1.5 wt%. This is due to the increased dielectric constant/capacitance of the doped PLA samples, in agreement with the dielectric layer doping techniques. For higher concentrations, the performance of the triboelectric generator deteriorated due to an increase in surface roughness resulting in reduced surface contact area between the triboelectric surfaces. For the ZnO-doped PLA, a continuous increase in the performance was observed as the doping concentration increased. This different behavior is attributed to the increased surface roughness of the SiC-doped PLA samples that results in a reduction in the active triboelectric surface area leading to a decreased triboelectric signal. In contrast, such behavior is not observed for the ZnO-doped PLA samples, where the surface roughness is not affected by ZnO doping.

## Figures and Tables

**Table 1:** Thickness measurements of the circular samples used for the capacitors.

Sample	Thickness (μm)	δThickness (μm)
0% ZnO	380	10.0
1% ZnO	385	10.0
2% ZnO	420	10.0
3% ZnO	415	10.0

## Data Availability

The data presented in this study are available on request from the corresponding author.
